# Major methylation alterations on the CpG markers of inflammatory immune associated genes after IVIG treatment in Kawasaki disease

**DOI:** 10.1186/s12920-016-0197-2

**Published:** 2016-08-12

**Authors:** Sung-Chou Li, Wen-Ching Chan, Ying-Hsien Huang, Mindy Ming-Huey Guo, Hong-Ren Yu, Fu-Chen Huang, Hsing-Chun Kuo, Ho-Chang Kuo

**Affiliations:** 1 Genomics and Proteomics Core Laboratory, Department of Medical Research, Kaohsiung Chang Gung Memorial Hospital and Chang Gung University College of Medicine, Kaohsiung, Taiwan; 2 Center for Research Informatics, The University of Chicago, Chicago, IL USA; 3 Kawasaki Disease Center, Kaohsiung Chang Gung Memorial Hospital, No.123, Dapi Rd, Niaosong Distict, Kaohsiung 83301 Taiwan; 4 Department of Pediatrics, Kaohsiung Chang Gung Memorial Hospital and Chang Gung University College of Medicine, No.123, Dapi Rd, Niaosong Distict, Kaohsiung 83301 Taiwan; 5 Department of Nursing, Chang Gung University of Science and Technology, Chiayi, Taiwan

**Keywords:** Kawasaki disease, DNA methylation, CpG marker, Intravenous immunoglobulin, Methylation alteration

## Abstract

**Background:**

Kawasaki disease (KD) is an autoimmune disease preferentially attacking children younger than five years worldwide. So far, the principal treatment to KD is the administration of Intravenous immunoglobulin (IVIG). Although DNA methylation plays important regulation roles in diseases, few studies investigated the regulation roles of DNA methylation in KD.

**Methods:**

In this study, we focused not only on the DNA methylation alterations resulted from KD onset but also on DNA methylation alterations resulted from IVIG administration. To do so, we investigated the effects of KD’s onset and IVIG administration on CpG marker’s methylation alterations.

**Results:**

We first found that DNA methylation alterations reflecting disease onset or IVIG administration are contributed mainly by the CpG markers on autosomes. In addition, we also demonstrated that some CpG markers carry methylation alteration among samples, forcing the expression abundance of the downstream genes to be also altered and negatively correlated with methylation profile. Finally, compared with KD’s onset, IVIG administration brings stronger impact on methylation alteration. And, such alterations were conducted mainly by hyper-methylating CpG markers, forcing the corresponding genes to keep lower expression levels. Moreover, the genes regulated by the altered CpG markers with IVIG administration are enriched in the pathways associated with inflammatory immune response.

**Conclusions:**

In summary, our result provides researchers with another way into the regulation mechanism through which IVIG represses excessive inflammatory responses.

## Background

Kawasaki disease (KD), also called mucocutaneous lymph node syndrome, is a childhood systemic vasculitis. It was first described by Dr. Tomisaku Kawasaki in 1967 [1]. KD mainly affects small to mediums sized vessels, with the development of coronary artery aneurysms being the most severe complication of KD. If left untreated, coronary artery lesions may develop in up to 25 % of patients with KD, which increases the risk of coronary artery aneurysm formation, coronary artery thrombosis, myocardial infarction or even sudden death [2, 3]. Current treatment guidelines for KD include intravenous immunoglobulin (IVIG) therapy during the acute phase. IVIG is given within the first ten days of fever and it may reduce the risk of coronary artery formation to 2–4 % [4]. In addition to treating KD, IVIG is also applied in treating patients with primary immune deficiency or acute infection by maintaining adequate level of IgG in circulation system and enhancing immune ability. However, in KD patients, IVIG represses excessive inflammation rather than enhances immune ability. Actually, there is still a debate on the precise mechanism by which IVIG represses immune response and that’s why we are curious about the methylation alteration after IVIG administration.

The exact pathogenesis of KD is still a matter of debate, although existing research points to both environmental and genetic factors. KD appears to be associated with ethnicity, and is more prevalent in Asian children [5]. Retrospective studies have also found that children whose parents or siblings were attacked by KD are at a higher risk of developing the disease [6, 7]. Genetic polymorphisms have also been identified in patients with KD, most of which have been associated with B cell and T cell immunity [8–10]. Apart from genetic factors, KD tends to occur in seasonal clusters suggesting an infectious or transmissible triggering agent [11].

DNA methylation plays an important role in regulation of gene expression through establishing and maintaining the DNA methylation status in gene promoters [12]. In mammals, within promoter regions, when the methyl group is chemically bounded to the cytosine of the CpG di-nucleotide (also called CpG marker), the expression abundance of the downstream gene is usually decreased. Therefore, by hypo- or hyper-methylating the CpG markers at the promoter regions, DNA methylation performs regulation abilities in diseases, especially when the downstream genes are functionally related to diseases [13–15].

In our previous study, we investigated the regulation roles of DNA methylation in KD by comparing the CpG markers’ methylation profiles between control and KD patients [16, 17]. We concluded that the CpG markers in FCGR2A promoter are hypo-methylated in KD patients, increasing the expression abundance of FCGR2A. In this study, we focused not only on the DNA methylation alterations resulted from KD onset but also on DNA methylation alterations resulted from IVIG administration. To do so, we collected DNA sample from control subjects, KD patients before IVIG treatment and KD patients after IVIG treatment. By bisulfite conversion of DNA samples and microarray detection of methylation profile, we investigated the effects of KD’s onset and IVIG administration on CpG marker’s methylation alterations.

## Methods

### Collection of subjects and clinical DNA samples

In Kaohsiung Chang Gung memorial Hospital, Taiwan, we enrolled 4 febrile control subjects (FC), 7 KD patients at acute phase (KD1) and 7 KD patients three weeks after IVIG treatment (KD3). Here, FC denotes febrile control, including the patients with fever but not diagnosed as KD by a physician. We collected whole blood samples from the subjects based on the IRB approvals by the Chang Gung Memorial Hospital (No.101-0680A3). Each KD3 subject was treated with IVIG (2 gm/kg) over a 12-hour period. Blood cells were collected and subjected to DNA extraction followed by bisulfite conversion of genomic DNA samples.

### Profiling of CpG methylation

We used Illumina HumanMethylation27 BeadChip to perform the genome-wide screening of DNA methylation patterns. HumanMethylation27 BeadChip was designed to detect methylation patterns of 27,578 CpG markers, spanning human genome. Detailed information of HumanMethylation27 BeadChip is available via the following URL: http://support.illumina.com/array/array_kits/infinium_humanmethylation27_beadchip_kit.html. For each HumanMethylation27 BeadChip assay, 200 ng of bisulfite-converted genomic DNA was applied according to the manufacturer’s instructions. For each CpG markers, HumanMethylation27 BeadChip designs two probes to distinguish cytosine (originally methylated) from thymine (originally un-methylated so that converted into uracil by bisulfite treatment and converted into thymine by reverse PCR reaction). By detecting the relative intensities of the probes for cytosine and thymine, methylation percentages (named β values) are determinable.

## Results and discussion

### Summary of significantly altered CpG markers on chromosomes

**Fig. 1:**
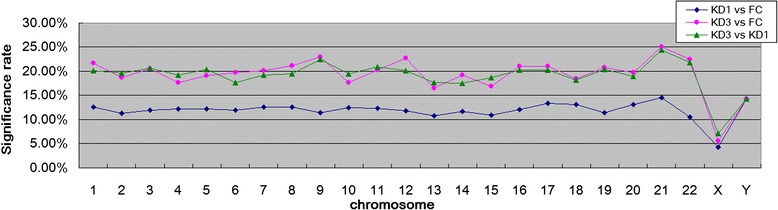
Distribution of significance rate along with chromosome. For each chromosome, we first tabulated the number of CpG markers on it. Then, the significance rate was defined as the ratio of the number of significantly altered CpG markers to the number of all CpG markers on the chromosome. The average significance rates for KD1 vs FC, KD3 vs FC and KD3 vs KD1 are 11.88, 19.30 and 19.13 %, respectively. In addition, the standard deviations for them are in order 1.91, 3.76 and 3.19 %. Significance was determined with *p* < 0.05 from *t*-test

In this study, we used Illumina HumanMethylation27 BeadChip to evaluate methylation patterns of CpG markers in FC (febrile control), KD1 (KD patients at the acute phase) and KD3 (KD patients three weeks after IVIG treatment) samples, comprising three comparisons namely KD1 vs. FC, KD3 vs. FC and KD3 vs. KD1. When the *p* < 0.05 criterion was set, we detected 3,249, 5,438 and 5,353 significantly altered CpG markers in KD1 vs. FC, KD3 vs. FC and KD3 vs. KD1 comparisons, respectively. For an overall understanding on the locations of significantly altered CpG markers on chromosomes, we investigated the distribution of significance rate, defined as the ratio of the number of significantly altered CpG markers to the one of all CpG markers on each chromosome. Figure 1 shows that the significance rate for KD1 vs FC comparison is evenly distributed on most chromosomes. In the other two comparisons, significance rate becomes significantly much higher and more diverse than the one in KD1 vs FC comparison. Figure 1 also demonstrates that fewer altered CpG markers distribute on X and Y chromosomes, implying that the DNA methylation alterations reflecting disease onset or IVIG treatment are contributed mainly by autosomes. Since higher methylation leads to lower gene expression, disease onset or IVIG treatment have little impact on the expressions of the genes sex chromosomes, especially on X chromosome.

### Major alterations in DNA methylation patterns caused by IVIG treatment

**Fig. 2:**
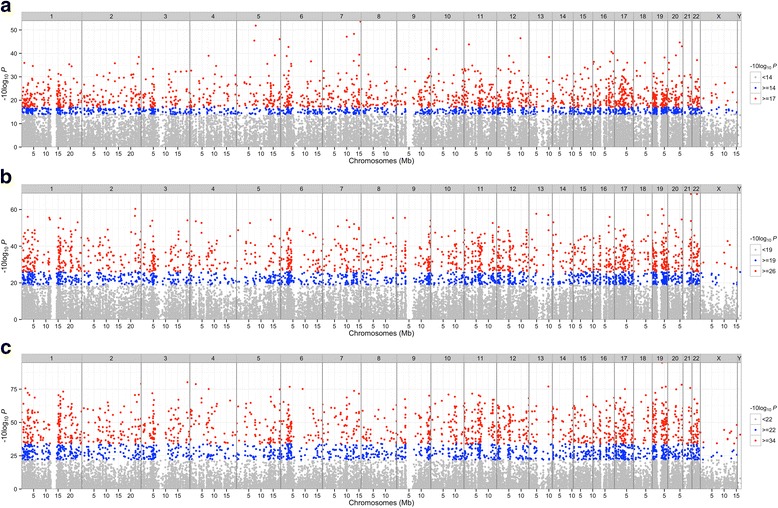
Manhattan plot of significant CpG markers. **a**, **b** and **c** belong to KD1 vs. FC, KD3 vs. FC and KD3 vs. KD1 comparisons, respectively. The ‘P’ in X axis denotes the p value of ANOVA analysis. Figure 2 shows that fewer CpG markers altered in KD1 vs. FC comparison than other two ones. The Manhattan plots were generated with R package ‘qqman’

**Fig. 3:**
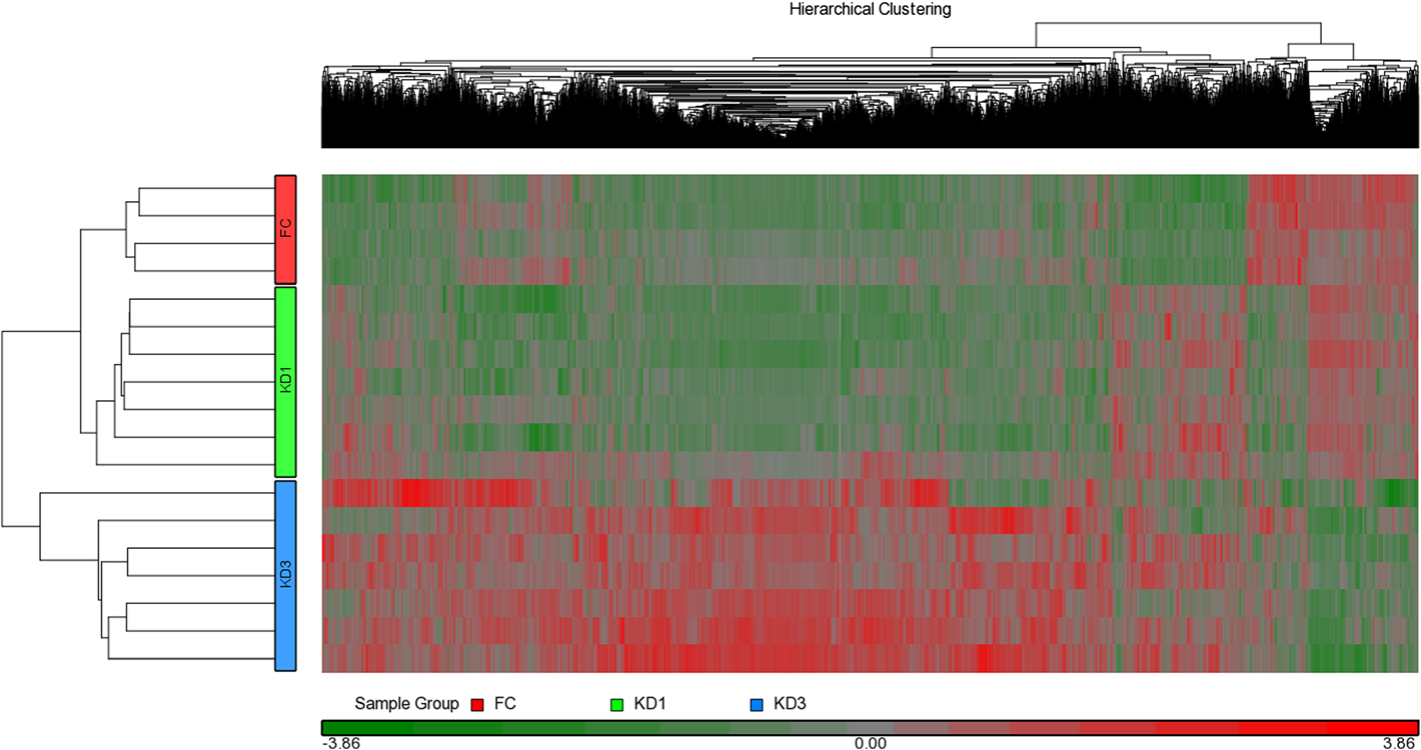
HeatMap of significantly altered CpG markers. By the union of three comparisons, including KD1 vs. FC, KD3 vs. FC and KD3 vs. KD1, there are totally 9,234 significantly altered CpG markers. The heatmap shows that methylation alteration caused by IVIG treatment is much more significant than the one caused by disease, which is consistent with the result in Fig. 2. Significance was determined with *p* < 0.05 from *t*-test

Next, we are interested in how significant the altered CpG markers are. Therefore, we generated a Manhattan plot for each comparison. As shown in Fig. 2, even with more stringent criteria (less p value), consistently, fewer CpG markers altered in the KD1 vs FC comparison than the other two ones, which implies that IVIG treatment causes major alterations in DNA methylation patterns. In addition, the effect of IVIG administration on the methylation alteration of CpG marker is much stronger than disease itself, which is proved by Fig. 3. In Fig. 3, the FC, KD1 and KD3 samples are exactly clustered based on their sample characteristics. In terms of the methylation patterns of CpG markers, FC and KD1 samples are pretty similar; while, KD3 samples carry much higher divergence. Figure 3 also shows that most significantly altered CpG markers are hypo-methylated in FC and KD1 sample; while, most cases in KD3 sample are hyper-methylated. Therefore, based on methylation regulation theory, in FC and KD1 samples, most genes regulated by the significantly altered CpG markers should be activated; while, the ones in KD3 samples should keep lower expression levels.

### Results of pathway enrichment analysis on the CpG marker related genes

IVIG is widely applied in autoimmune disease, including KD, to repress harmful inflammation [18]. To understand what genes are regulated by the significantly altered CpG markers with IVIG administration, we derived the genes nearby the markers followed by pathway enrichment analysis on the genes. From a list of input genes, pathway enrichment analysis tries to identify the pathways significantly enriched by the input genes based on a hypergeometric test. And, pathway enrichment analysis provides more informative data, enabling the researchers consider the genes in a functional unit rather the individual gene [19]. After pathway enrichment analysis, a list of significant pathways were generated from the input genes in both KD3 vs FC and KD3 vs KD1 comparisons. We took the intersection of the two lists, presenting the pathways in Table 1.

**Table 1:** The results of pathway enrichment analysis. We did pathway enrichment analysis with Partek on the genes located by the CpG markers significantly altered in KD3 vs FC or KD3 vs KD1 comparisons. Among the significant pathways (*p* < 0.05), four are co-enriched in the two comparisons

Pathway name	*p*-value: KD3 vs FC	*p*-value: KD3 vs KD1
Hematopoietic cell lineage	4.10E-10	7.50E-09
Cytokine-cytokine receptor interaction	2.59E-09	9.52E-12
Chemokine signaling pathway	0.00246012	2.68E-05
Jak-STAT signaling pathway	0.00287132	0.000381639

Table 1 demonstrates that the significant pathways shared by the two comparisons are Hematopoietic cell lineage, Cytokine-cytokine receptor interaction, Chemokine signaling pathway and Jak-STAT signaling pathway. Through the hematopoietic cell lineage pathway, hematopoietic stem cell differentiates into different blood cells, including T cell, NK cell, basophil, macrophage, B cell and so on, responding to diverse stimulus or necessity [20]. Therefore, hematopoietic cell lineage plays important roles in immune response. Cytokine are soluble proteins, secreted by donor cells responding to stimulus and transported by circulation system to target cells. Through the cytokine-cytokine receptor interaction pathway, cytokines bind to their receptors on the target cell membrane [21]. Next, through the Jak-STAT signaling pathway, the external signals initiated by cytokines can be passed down into the target cells, triggering immune activities and activating inflammation responses [22]. Similar to cytokine, chemokine also plays positive roles in up-regulating inflammatory immune responses [23]. Therefore, the pathways altered by IVIG administration are immune-related. They play either up-stream or down-stream roles in regulating inflammatory immune response.

Actually, there is still a debate on the precise mechanism by which IVIG represses immune response, although several possible mechanisms have been proposed [24–28]. Our results of pathway enrichment analysis shows that IVIG administration performs the ability of repressing harmful inflammation through hypo- or hyper-methylating the CpG markers whose down-stream genes are involved inflammatory immune response. Such result provides the researchers with another way into the regulation mechanism through which IVIG represses excessive inflammatory responses. Although our result provides researchers with another way into the regulation mechanism through which IVIG represses excessive inflammatory responses, we can not exclude the involvement of aspirin. Salicylic acid is the major compound of aspirin and is usually administrated with IVIG for alleviation of excessive inflammatory immune response. Therefore, the effect of salicylic acid on methylation alteration needs more attentions.

### Methylation alteration between samples

**Fig. 4:**
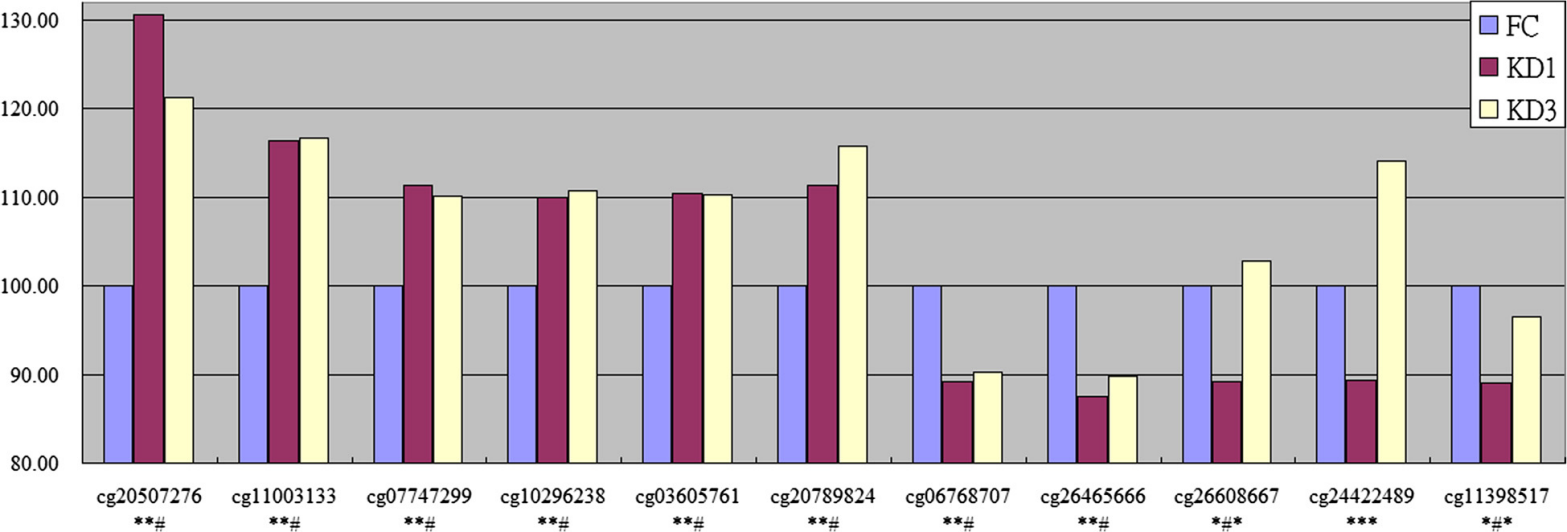
Methylation alteration in comparisons. There are 11 CpG markers that are significantly altered (*p* < 0.05, *t*-test) and carry more than 10 % alteration in at least two of the three comparisons. For each CpG marker, the average methylation percentage in FC samples was set to 100 %. The relative average methylation percentages in KD1 and KD3 samples were plotted. For cg20507276, the first two ‘*’ markers denote significant alterations in KD1 vs. FC and KD3 vs. FC comparisons. The final ‘#’ mark denotes un-significant alteration between KD3 vs. KD1 comparison

Figure 3 also shows that a small fraction of CpG markers are characterized with altered methylation patters between samples. Figure 4 illustrates 11 CpG markers that are significantly altered (*p* < 0.05) and carry more than 10 % alteration in at least two of the three comparisons. The first six CpG markers are hyper-methylated in KD1 samples when compared with FC samples where the average methylation percentages are set to 100 %. So, the onset of KD increases the methylation degree on these markers. However, with the administration of IVIG, these markers did not significantly recover from the hyper-methylated status. Another kind of CpG markers with altered methylation patters are the last 5 markers that are hypo-methylated in KD1 samples when compared with FC ones. So, the onset of KD decreases the methylation degree on these markers. Moreover, with the administration of IVIG, two markers (cg06768707 and cg26465666) sticks to hypo-methylation status. Meanwhile, the last three markers recover from the hypo-methylated status, keeping no significant alteration from FC samples.

### Altered change of gene expression between samples

**Fig. 5:**
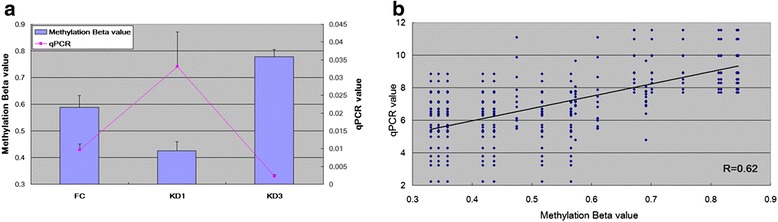
Integration of methylation pattern and gene expression profile for FCGR2A. The CpG marker cg24422489 is located at the promoter region (244 nt. in front of the transcription start site) of FCGR2A. **a** The methylation pattern of cg24422489 and gene expression profile of FCGR2A are negatively correlated. In addition, they are altered in FC, KD1 and KD3 samples. For qPCR curve, the X axis unit is the expression ratio of FCGR2A to the internal control gene, 18S. Histogram and curve are presented as mean + SEM. **b** For an un-biased result, we did 10,000 re-sampleings individually in FC, KD1 and KD3 sets followed by deriving the Pearson’s correlation coefficient. The result shows that methylation pattern is positively correlated with ΔCt values. Therefore, hyper-methylation represses gene expression. The qPCR result is from the previous study [16]

DNA methylation on the CpG markers at genes’ promoters are thought to decrease the expressions of the downstream genes. Therefore, the methylation alteration of CpG markers implies altered reversal change of gene expression between samples. To investigate whether negative correlation exists between DNA methylation and gene expression or not we did further analysis on FCGR2A gene which was reported to be a susceptibility locus for KD [29, 30] and carries the CpG marker, cg24422489, at its promoter region (-224 to the transcription start site). As shown in Figs. 4 and 5a, in KD samples, cg24422489 demonstrates a hypo-methated status which was subsequently eliminated with the hyper-methylation by the administration of IVIG. On the contrary, the expression level of FCGR2A demonstrates reverse tendency between samples. Such result was summarized based on 18 methylation assays and 60 qPCR experiments (26 on FC, 24 on KD1 and 10 on KD3 samples). For a more un-biased estimate, we did re-sampling assay. For FCGR2A’s methylation β value and ΔCt value, we did 10,000 runs of Bootstrap re-sampleing [31, 32] individually in FC, KD1 and KD3 sets followed by calculating the Pearson’s correlation coefficient of the 30,000 data points. The result shows that methylation pattern is positively correlated with ΔCt values (R = 0.62). Therefore, hyper-methylation at gene promoter represses gene expression.

It is a consensus that hypermethylation at gene promoter decreases the expressions of the downstream gene. However, not every hypermethylation brings down-regulation of genes. Such cases can also be observed in our data. Actually, gene expression undergoes several regulation mechanisms, including acetylation of chromatin [33], methylation at CpG marker, the binding of transcription factor on promoter [34], miRNA regulation [35] and so on. Therefore, gene expression abundance is an overall result of all regulation mechanisms. So far, it is difficult to monitor all regulation mechanisms simultaneously so that only methylation at CpG marker is considered in this study.

## Conclusions

We first found that DNA methylation alterations reflecting disease onset or IVIG administration are contributed mainly by autosomes; sex chromosomes only contribute little. In addition, some markers carry methylation alteration between samples. As a result, their downstream genes are also altered and are negatively correlated with methylation profile. Finally, IVIG administration brings stronger impact on methylation alteration than KD onsets does. And, such alterations were conducted mainly by hyper-methylating CpG markers. Moreover, the genes whose promoter CpG markers are altered with IVIG administration are enriched in the pathways associated with inflammatory immune response.
